# Frontiers of biology in human diseases: strategies for biomolecule's discovery, nanobiotechnologies and biophotonics

**DOI:** 10.1186/1753-6561-8-S4-O9

**Published:** 2014-10-01

**Authors:** Luiz Goulart, Noelio Dantas, Anielle Silva, João Marcos Madurro, Ana Graci Brito-Madurro, Carlos Ueira-Vieira, Patricia Fujimura, Yara Maia, Paula Santos, Ana Paula Freschi, Juliana Almeida, Isabela Goulart

**Affiliations:** 1 Federal University of Uberlandia, Institute of Genetics and Biochemistry, and University of California Davis, Dept of Medical Microbiology and Immunology, CA, USA; 2 Federal University of Uberlandia, Institute of Physics, Uberlândia, Brazil; 3 Federal University of Uberlandia, Institute of Chemistry, Uberlândia, Brazil; 4 Federal University of Uberlandia, Institute of Genetics and Biochemistry, Uberlândia, Brazil; 5 Federal University of Uberlandia, School of Medicine, National Reference Center on Leprosy and Dermatological Diseases, Uberlândia, Brazil

## Introduction

The current frontiers in biological sciences demand an interface among disciplines of biology, chemistry, and physics to achieve new paradigms in applied nanobiotechnologies to health. An extraordinary amount of information on genomes, transcriptomes, proteomes, metabolomes, miRNAs, lncRNAs, protein editing, post-translational modifications, exosomes, molecular interactions, cell signaling, structural biology, immunology, cellular receptors, interactomes, and bioinformatics are opening new possibilities for biological manipulations aiming improved diagnostics and therapeutics. New rationale is required to use available technologies that intersect among imaging, electrochemistry, biophotonics, nanotechnologies and combinatorial molecules. A massive, diverse and broad knowledge on multidisciplinary aspects have been provided in the last few years, and the challenge is to combine recent technologies and information to identify revolutionary platforms for the progression of life sciences. This brief review will discuss examples of epitope-based and combinatorial antibody and nucleic acid (aptamers) selection technologies in association with nanobiotechnologies and their multiple applications in biomedical sciences.

## Discovery of biomarkers using combinatorial technologies

Combinatorial libraries displaying very diverse set of random peptides or very large repertoire of antibody fragments' fused to the capsid surface of filamentous phage have been successfully exploited in the discovery of novel biomarkers, and are excellent platforms for the design of high-affinity, protein-based binding reagents. Such markers are either peptide ligands that mimic epitope regions (mimotope) or monoclonal antibody fragments (Fab or scFv) against specific targets selected through cycles of biopanning (binding-washing-elution-transformation-amplification), also called Phage Display. The fundamental advantage of this technology is the direct link between the experimental phenotype and its encapsulated genotype, which allows the evolution of selected binders into optimized molecules [1,2]. Peptide ligands may be used to map immunologically active sites in proteins, carbohydrates, lipids, and also as individual epitope-mimicking antigens and immunogens [3,4]. The selected mimotopes may also be directly used in phage-based ELISA immunoassays, resulting in simple, specific, sensitive, and low-cost immunodiagnostic tests [5]. Similarly, the selection of antibodies from combinatorial libraries has also become an important tool for the generation of reagents, diagnostics, and therapeutic molecules. It is the only method to obtain specific antibodies bypassing the immunization step, which mimics the maturation process of human antibody *in vivo*, resulting in high affinity antibody ligands, which may be suitable to human administration and potentially applicable to clinical diagnosis and treatment. We have generated successful molecules for diagnostic [5-8] and therapeutic [7,9] purposes through phage display by *in vitro, ex vivo *and *in vivo *approaches of selection. Such strategies are dependent on a very stringent subtractive selection scheme that includes well-characterized target samples and controls, target purification, elution/washing protocol with or without competitor molecules, number of selection cycles, and quality of the library and its diversity.

Another important combinatorial technology is the selection of aptamers, which are random RNA or DNA sequences with conformational actions that specifically bind to a variety of targets, including proteins, small molecules, oligonucleotides, and peptides. In RNA aptamer selections, a DNA pool containing random sequences is typically synthesized artificially, which are subsequently transcribed to give the starting RNA population. Aptamer ligands are then evolved during several cycles of selection, elution, PCR amplification, cloning and transformation. Among several advantages of aptamers over other common biomarkers, we can include the similar molecular recognition of antibodies, the ability to be completely engineered in test tubes, the chemical synthesis at very low costs, desirable storage features due to their resistance to high temperatures and humidity without degradation, lack of immunogenicity, high potency and specificity. We have successfully selected aptamers for prostate cancer diagnosis, and to validate its potential use, magnetic beads were functionalized with our novel aptamer that promptly recognized circulating tumor cells, and can be an auxiliary tool for molecular diagnostics.

Briefly, short peptides, antibody fragments and aptamers aiming the selection of functional core sequences of epitope or paratope targets can be used in combination with nanotechnologies and biophotonics for improvement of diagnostic platforms, which will be further explored.

## Electrochemistry

Biosensors combine the biological nature of markers with the power of microelectronics and optoelectronics to offer powerful analytical tools with major applications in medical diagnostics. The foundations of biological sensors' technology involve the interplay of fundamental disciplines, demanding specific knowledge on physical chemistry (nanoparticles), materials science (polymers), physics (optics and solid state), biology (antigen, antibody, biochemistry, genetics, substrates, and clinical information of diseases) and engineering (electronics and microfabrication). The specific interaction between the target analyte and the complementary recognition layer functionalized with a biomarker produces a physico-chemical change that is detected and measured by an electrochemical transducer through electric alterations as voltage (voltammetry), current (amperometry) or resistance (impedance). We have developed specific biosensors for nucleic acids and proteins detection using electrochemistry technology for dengue virus, leishmaniasis, leprosy, breast and prostate cancer, and many other diseases [8,10]. Our aim is to translate these parameters into a universal platform using specific polymers as electrodes that not only allow prompt conjugation of biomarkers, but also mediate electron transfer kinetics to achieve a faster response in a simple, portable, specific and sensitive instrument. However, other technologies are also very competitive in the diagnostic field, due to specific functionalities and uses, such as target capture and enrichment by magnetic nanoparticles [10], imaging mediated by quantum dots, and other biophotonic technologies for the improvement of conventional assays, which is further discussed.

## Nanoparticles and biophotonics

Microparticle agglutination test has been used in clinical diagnostics since 1980's. However, nanoparticles only recently became important diagnostic tools due to the development of different synthesis routes, chemical groups and polymers. Our first approaches with nanoparticles were focused on agglutination assays, specifically with colored-latex Particle Gel Immunoagglutination test (PaGIa) and Magnetic Microparticle-ELISA (MME) for antigen and antibody detection. The agglutination tests were performed with microparticles and nanoparticles coupled with filamentous bacteriophages displaying fused mimotopes on its surface, which has favored the formation of the antigen-antibody or peptide-protein complexes, amplifying the optical detection in ELISA assays or after the chromatographic separation of the microagglutinates [10].

Different from latex nanoparticles, a new class of semiconductor nanocrystals, called Quantum Dots (QDs) has been developed with potential applications in diagnosis, drug delivery, biomedical imaging, sensors and optoelectronics. However, QDs are still considered toxic due to the use of hazardous reagents and synthesis under high temperatures, which affect their stability and biocompatibility. These QDs are highly luminescent and their emission wavelengths are dependent on their diameter, varying from 2 to 20 nm. Recently, our group has synthesized a novel class of ultrasmall QDs through an aqueous phase route (green chemistry), also called magic-sized QD. These extremely small nanocrystals present diameters below 2 nm, are highly stable, non-toxic, non-immunogenic, and with proper functionalization may become a powerful biomarker that may be used in many different applications, including immunohistochemistry and *in vivo *imaging studies, as shown with a breast cancer cell line.

Interestingly, other biophotonic technologies have also the potential to be used in clinical and laboratory settings. We have explored the surface plasmon resonance (SPR) technology to improve the conventional ELISA tests of many different diseases. Surface plasmons are surface electromagnetic waves that propagate in a direction parallel to the metal/dielectric interface, usually formed with gold nanofilms. The wave is on the boundary of the gold polymer with the external medium (sample), and these oscillations are very sensitive to any changes of this boundary, such as the adsorption of molecules to the functionalized gold surface. The technology relies on reflectivity measurements used to detect DNA or proteins by changes in the local index of refraction upon adsorption of the target molecule to the functionalized gold surface without labeling. We have reached very high sensitivity using specific phage display-derived biomarkers for leprosy and dengue virus detection, which are diseases with diagnostic problems due to their nature and specific clinical conditions, and results indicate that simple SPR systems can substitute conventional ELISA tests in the near future.

## Conclusion and perspectives

We have successfully shown that combinatorial technologies have revealed functional determinant sites of molecules, which were combined with multiple research tools, techniques, and instruments that enabled entirely novel approaches for diagnostics and therapeutics.

We believe that extensive and global analytical techniques, such as genomics and proteomics, may allow major target identification, but cross-reactions still remain as a problem, which can be solved by reducing the target site, a goal that has been promptly achieved by Phage display or Aptamer selections.

The search for universal and robust diagnostic platforms and for highly specific and sensitive markers have been the highest challenges in the medical field, due to the variable disease spectra, different pathogenetic backgrounds, specific sampling, the complex interactions with vectors and environments, which may result in very diverse phenotypes [10]. Only multidisciplinary teams and novel nanobiotechnological approaches can meet the demand of smart solutions for human diseases diagnostics.
